# Association between the rs7903146 Polymorphism in the *TCF7L2* Gene and Parameters Derived with Continuous Glucose Monitoring in Individuals without Diabetes

**DOI:** 10.1371/journal.pone.0149992

**Published:** 2016-02-25

**Authors:** Sabrina van der Kroef, Raymond Noordam, Joris Deelen, Abimbola A. Akintola, Steffy W. M. Jansen, Iris Postmus, Carolien A. Wijsman, Marian Beekman, Simon P. Mooijaart, P. Eline Slagboom, Diana van Heemst

**Affiliations:** 1 Department of Gerontology and Geriatrics, Leiden University Medical Center, Leiden, the Netherlands; 2 Section of Molecular Epidemiology, Department of Medical Statistics and Bioinformatics, Leiden University Medical Center, Leiden, the Netherlands; Virgen Macarena University Hospital, School of Medicine, University of Seville, SPAIN

## Abstract

**Background:**

The rs7903146-T allele in the transcription factor 7-like 2 (*TCF7L2*) gene has been associated with impaired pancreatic insulin secretion, enhanced liver glucose production, and an increased risk of type 2 diabetes. Nevertheless, the impact of rs7903146 on daily glucose trajectories remains unclear. Continuous glucose monitoring (CGM) can estimate glycemia and glycemic variability based on consecutive glucose measurements collected over several days. The purpose of the present study was to investigate the associations of rs7903146 with glycemia and glycemic variability in middle-aged participants without diabetes.

**Methods:**

Complete data from 235 participants without diabetes from the Leiden Longevity Study were available. Participants were divided into two groups based on rs7903146 genotype; rs7903146-CC genotype carriers (N = 123) and rs7903146-CT/TT genotype carriers (N = 112). Validated parameters of glycemia (e.g., mean 24h glucose level) and glycemic variability (e.g., 24h standard deviation) were derived from data collected with a CGM system for a 72-hour period.

**Results:**

The study population was on average 64.7 years old (standard deviation = 5.9) and composed of 49.8% of women. Compared with rs7903146-CC carriers, rs7903146-CT/TT carriers exhibited a trend towards a higher mean 24-hour glucose level (5.21 versus 5.32 mmol/L; p-value = 0.15) and a significantly higher mean nocturnal glucose (3:00am– 6:00am; 4.48 versus 4.67 mmol/L; p-value = 0.03) that was explained for 34.6% by body weight and percentage body fat. No differences in measures of glycemic variability between the genotype groups were observed.

**Conclusion:**

Despite limited sample size, our study indicates that the rs7903146-T allele in *TCF7L2* was associated with a higher mean nocturnal glucose dependent on body composition, which might suggest that rs7902146 affects liver-specific aspects of glucose metabolism.

## Introduction

The transcription factor-7-like-2 (*TCF7L2*) gene has been consistently associated with type 2 diabetes mellitus (T2D) [1] in different ethnic groups [2,3], and with fasting glucose levels [4]. The *TCF7L2* gene encodes the transcription factor 4 (TCF4) which is involved in Wnt signalling [5]. In the nucleus, stabilized β-catenin binds to TCF transcription factors to regulate the transcription of Wnt target genes [6]. The Wnt/β-catenin signalling pathway affects pancreatic β-cell development and function, and thus affects glucose metabolism [7,8].

In genome-wide association studies (GWAS), the rs7903146-T allele located in an intronic region of the *TCF7L2* gene has been associated with a higher risk of T2D [9–11] via mechanisms other than higher/lower *TCF7L2* mRNA expression levels [12,13]. Also, this allele has been associated with impaired β-cell function [14] and impaired insulin secretion [7]. This suggests that the T allele of rs7903146 may increase the risk of T2D via effects on insulin secretion [15]. However, the rs7903146-T allele has also been associated with an enhanced rate of hepatic glucose production [7], which may suggest different mechanisms by which rs7903146 polymorphism could affect the risk of T2D.

Glycemic variability is emerging as a risk factor for complications, mainly microvascular-related, in T2D patients [16]. Therefore, continuous glucose monitoring (CGM) is increasingly used in routine clinical practice for T2D patients. It is a minimally invasive method to determine glucose levels from interstitial fluid via a glucose sensor that is usually implanted in the abdominal subcutaneous tissue. In this way, parameters of glycemia and of glycemic variability, including mean diurnal and nocturnal glucose levels can be evaluated during normal activities of daily living [17]. However, although in individuals without diabetes postprandial glucose excursions into the hyperglycaemic range as well as nocturnal hypoglycemia have been observed, the clinical meaning has yet to be determined [18]. Hence, the CGM system may be useful to determine whether daily glucose trends are affected by the rs7903146 polymorphism. In this study, our purpose was to investigate the association between rs7903146 in *TCF7L2* and CGM derived measures in a cohort of middle-aged participants without diabetes.

## Methods

### Ethical statement

The Medical Ethical Committee of the Leiden University Medical Centre approved this study. Written informed consent was obtained from all study participants.

### Study setting

The present study was embedded in the Leiden Longevity Study. This study originally aimed to investigate biomarkers and genetic variation associated with familial longevity. A more detailed description of the design and recruitment strategy of the Leiden Longevity Study has been published previously [19]. In short, a total of 421 long-lived families were recruited, without selection based on health condition or demographics. Families were included when at least two long-lived siblings were still alive and fulfilled the age criteria of 89 years for men and 91 years for women. In total, 1671 offspring of these long-lived individuals were recruited. Furthermore, a total of 744 partners thereof were recruited as controls.

### Study population

A subsample of 235 participants (offspring and controls) of the Leiden Longevity Study had data available on rs7903146 genotype and measures derived with continuous glucose monitoring. Participants with diabetes mellitus (type 1 or 2) were not invited to participate nor were participants with a body mass index (BMI) lower than 19 kg/m^2^ and higher than 33 kg/m^2^.

### Genotyping

Rs7903146 was extracted from whole genome data. Genotyping was conducted with the Illumina Human 660W-Quad and OmniExpress BeadChips (Illumina, San Diego, CA, USA). Individuals were excluded from further investigation if they had a mismatch in sex or familial relatedness based on genotype and phenotype. The allele frequency of rs7903146 was comparable with what is observed in other Caucasian populations [1], and the genotype distribution was in Hardy-Weinberg equilibrium (p-value > 0.05). For sample size issues, we combined participants carrying the rs7903146 CT genotype with those carrying the TT genotype.

### Continuous Glucose Monitoring

The Mini-Med^®^ CGM System (Medtronic MiniMed In., Northridge, CA) was used by all participants included in this project. A glucose sensor (Sof-Sensor^®^, Medtronic Minimed Inc., Northridge, CA) was inserted into the subcutaneous abdominal fat tissue to monitor glucose levels of interstitial fluid every 5 minutes for five consecutive days. To calibrate the sensor, participants were asked to measure capillary blood glucose by finger prick four times a day. Participants were encouraged to pursue their normal daily activities while wearing the glucose monitor. The participants were asked to register food intake, medication intake and physical exercise in a logbook. In line with the guidelines from the manufacture, we excluded the first and fifth day of the measurement, as these were considered least accurate.

### Calculations

Of the data collected by the CGM system, multiple parameters were calculated, including overall mean 24-hour glucose level, mean nocturnal glucose level (3:00am– 6:00am) and mean diurnal glucose level (6:00am– 0:00am). Additionally, we calculated parameters of glycemic variability, including 24-hour standard deviation (SD), the continuous overlapping net glycemic action (CONGA 4), and the mean of daily differences (MODD) for every participant separately [20]. The CONGA 4 determines intraday glycemic variability. For each observation after the first four hours of observations, the difference between the current observation and the observation four hours earlier was calculated. The CONGA 4 was defined as the standard deviation of these differences [21–23]. The mean of daily differences (MODD) was determined to evaluate interday variability. The MODD represents the mean of the absolute differences of glucose values obtained at exactly the same time of day, from two consecutive days. The calculated measures of glycaemia and glycemic variability have been used before in other studies [16,17,24], and have been validated [25].

### Biochemical analyses

Fasting serum morning samples were taken from all participants to measure the levels of glucose and insulin. All measurements were conducted with fully automated equipment from Roche Diagnostics (Almere, the Netherlands; coefficients of variation <7.5% for glucose and <6.8% for insulin). All measurements were performed at the Department of Clinical Chemistry and Laboratory Medicine, Leiden University Medical Centre, Leiden, the Netherlands.

### Anthropometrics

For all participants, height, weight, waist and hip circumference were measured at the study center. BMI was calculated as weight in kilograms divided by height in meters squared. Waist to hip ratio (WHR) was calculated by dividing waist circumference by hip circumference. Percentage of body fat (PBF) was determined according to a mobile Bioelectrical Impedance Analysis (BIA) system (Bodystat^®^ 1500 Ltd, Isle of Man, British Isles).

### Statistical analysis

Study characteristics were studied for the whole population, as well as separately for participants carrying the rs7903146-CC and rs7903146-CT/TT genotype.

We studied the associations between rs7903146 and measures of glycemia (e.g., nocturnal glucose) and glycemic variability (e.g., 24h SD and CONGA 4) by comparing these measures between the rs7903146-CC and rs7903146-CT/TT carriers. The comparisons were statistically tested using linear regression models in STATA v12.0 (StataCorp LP, College Station, Texas, USA). All statistical analyses were adjusted for age, sex, and offspring/partner status. We used the clustered robust option, which clusters related participants in the analyses, in the linear regression model in STATA to correct for familial relationships between the participating offspring in the study. P-values for these analyses were additionally obtained through Monte Carlo permutation tests (1000 times) in STATA.

In a separate analysis, we additionally statistically adjusted the comparisons of the CGM measures for the study characteristics that reached the level of statistical significance in the comparison between carriers of the rs7903146-CC and rs7903146-CT/TT genotype. This was conducted to study whether any of the observed differences between the rs7903146-CC and rs7903146-CT/TT carriers in CGM measures were mediated by any of the study characteristics. Before the mediation analyses, we tested for multiplicative interaction between rs7903149 carriership and the study characteristics. In case the multiplicative interaction did not reached statistical significance, the percentage explained by these additional variables was calculated, as is explained in more detail elsewhere [26].

Two-sided p-values below 0.05 were considered statistically significant.

## Results

### Study characteristics

Table 1 describes the characteristics of the total study population, as well as stratified for CC carriers (N = 123) and CT/TT carriers (N = 112) of rs7903146. The total study population had a mean age of 64.7 years (standard deviation [SD] = 5.9), comprised for 49.8% of females, and comprised for 62.6% of offspring. Compared with rs7903146 CC-genotype carriers, carriers of the CT/TT genotype had a higher body weight (79.2 versus 76.7 kg; p-value = 0.03) and a higher percentage of body fat (30.3% versus 31.8%; p-value = 0.02). None of the other study characteristics were significantly different between the two study groups, although rs7903146 CT/TT-genotype carriers tended to have a higher waist-to-hip ratio (p-value = 0.06) and BMI (p-value = 0.08).

**Table 1:** Characteristics of the study population.

		Stratified by rs7903146 carriership		
	Total	CC	CT/TT	
Characteristics	(N = 235)	(N = 123)	(N = 112)	p-value
**Demographics**				
Age, years	64.7 (5.9)	64.5 (0.6)	64.8 (0.6)	0.68
Females, n (%)	117 (49.8)	55 (44.7)	62 (55.4)	0.14
Offspring of long-lived siblings, n (%)	147 (62.6)	83 (67.5)	64 (57.1)	0.17
**Anthropometrics**				
Length, cm	172 (8.7)	172 (0.6)	173 (0.6)	0.37
Body weight, kg	77.9 (12.3)	76.7 (1.0)	79.2 (0.9)	**0.03**
Body mass index, kg/m^2^	26.2 (3.4)	25.8 (0.3)	26.6 (0.3)	0.08
Percentage of body fat, %^#^	31.0 (8.3)	30.3 (0.5)	31.8 (0.5)	**0.02**
Waist Hip Ratio^#^	0.92 (0.08)	0.91 (0.01)	0.93 (0.01)	0.06

Values of the total population are expressed as mean (standard deviation), unless indicated otherwise. For the values stratified by rs7903146 carriership, data was presented as the mean (standard error of the mean). Analyses adjusted for age, sex and offspring/partner relationship. Analyses were corrected for familial structure using robust standard error.

^#^Data available for n = 210 (representing 115 CC carriers and 95 CT/TT carriers).

### Association between rs7903146 and CGM system outcomes

**Fig 1:**
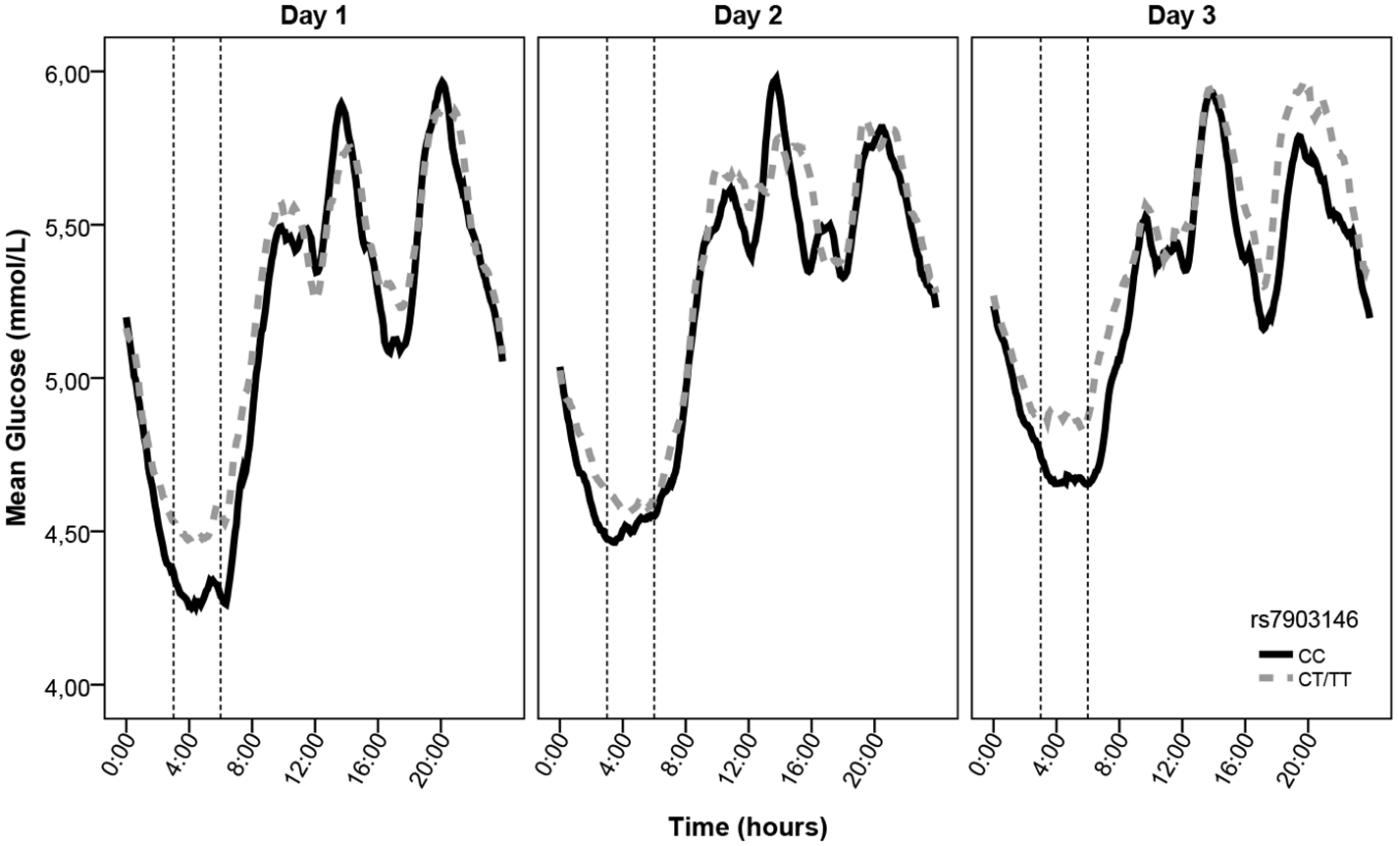
Glucose rhythm over the three days, stratified per genotype group. Mean glucose rhythms during 72 hours for carriers of the protective alleles (black line) and the risk alleles (grey line) of rs7903146 in *TCF7L2*. The time span between the dotted black lines (3.00–6.00h) represents the nocturnal hours during which people are (on average) fast asleep.

Fig 1 presents a graphical representation of the average glucose rhythm during 72 hours separately for CC genotype carriers and CT/TT genotype carriers. Specifically, the mean level of nocturnal glucose tended to be higher in carriers of the CT/TT genotype.

Table 2 presents fasting venous glucose and insulin levels and parameters derived from the CGM system (glycemia and glycemic variability) separately for the CC genotype and CT/TT-genotype carriers of rs7903146. Compared with rs7903146-CC carriers, carriers of CT/TT had a similar mean level of fasting glucose (5.14 versus 5.23 mmol/L; p-value = 0.33). However, rs7903146-CT/TT carriers tended to have higher levels of fasting insulin than in CC carriers (2.29 versus 2.10 mU/L), although not significant (p-value = 0.11). With respect to CGM-derived measures, we observed that carriers of the CT/TT genotype had a significantly higher mean level of nocturnal glucose (between 3:00am– 6:00am) compared to CC genotype carriers (4.67 versus 4.48 mmol/L; p-value = 0.02). Mean levels of diurnal glucose and the mean 24-hour glucose level also tended to be higher in CT/TT genotype carriers than in CC genotype carriers, but these were not statistically significant. We observed no differences in the measures of glycemic variability between the two groups. Furthermore, results from the Monte Carlo permutation test were similar with respect to the normal regression analysis.

**Table 2:** Fasting and Continuous Glucose Monitoring parameters over 72-hour period for carriers of the protective (CC) and risk (CT/TT) alleles of rs7903146 in *TCF7L2*.

	CC	CT/TT	p-value^##^	p-value^$$^
	(n = 123)	(n = 112)		
**Fasting parameters (venous)**				
Glucose, mmol/L^#^	5.14 (5.02–5.26)	5.22 (5.11–5.33)	0.33	0.37
Insulin, mU/L^$, †^	2.11 (1.99–2.24)	2.27 (2.12–2.44)	0.11	0.07
**Continuous Glucose monitoring**				
**Glycaemia, mmol/L**				
24-hour mean glucose	5.21 (5.13–5.30)	5.32 (5.22–5.42)	0.15	0.08
Nocturnal glucose (3.00h-6.00h)	4.48 (4.38–4.59)	4.67 (4.55–4.79)	**0.03**	**0.04**
Diurnal glucose (6.00h-0.00h)	5.40 (5.31–5.49)	5.50 (5.39–5.60)	0.21	0.14
**Glycemic variability**				
24h SD	0.92 (0.87–0.97)	0.92 (0.87–0.97)	0.95	0.93
CONGA4	1.16 (1.08–1.23)	1.16 (1.09–1.22)	0.97	0.92
MODD	0.85 (0.80–0.90)	0.86 (0.81–0.91)	0.76	0.70
Range	4.92 (4.64–5.20)	5.00 (4.72–5.27)	0.71	0.73

Abbreviations: CONGA4, Continuous net glycemic action; MODD, Mean of daily difference; SD, standard deviation. Data reported depict the estimated (geometric) means with the 95% confidence interval. Analyses adjusted for age, sex and offspring/partner relationship. Analyses were corrected for familial structure using robust standard error.

^#)^ Data available for n = 161 (representing 85 CC carriers and 76 CT/TT carriers).

^$)^ Data available for n = 206 (representing 113 CC carriers and 93 CT/TT carriers).

^†)^ Geometric means due to skewness of data.

^##)^ P-value obtained from the linear regression model.

^$$)^ P-value obtained after 1000 permutations.

In the subsample of participants with data available on percentage of body fat and body weight (the two study characteristics significantly different between CC- and CT/TT-rs7903146 carriers), CT/TT carriers had a 0.17 mmol/L (standard error = 0.09) higher nocturnal glucose than CC carriers, which was marginally not statistically significant (p-value = 0.08). We observed no multiplicative interaction between rs7903146 and the measures of body composition on nocturnal glucose levels (p-values >0.05). Additional adjustment for percentage of body fat and body weight gave a difference between CC and CT/TT carriers of 0.11 mmol/L (standard error = 0.10) in mean nocturnal glucose level, which was not statistically significant (p-value = 0.26). Based on these numbers, estimated mediation by body weight and percentage of body fat was 34.6%.

## Discussion

We aimed to investigate the association between rs7903146 in *TCF7L2* and measures of glycemia and glycemic variability derived with CGM. While rs7903146 was not associated with the investigated measures of glycemic variability, we observed that rs7903146-CT/TT carriers, who have a higher T2D risk [9–11], have a higher mean nocturnal glucose level than rs7903146-CC carriers. This association was partly mediated by measures of body composition.

Previously, the rs7903146-T allele has been associated with higher fasting glucose levels in blood [4]. Nocturnal glucose might reflect similar aspects of glucose regulation as fasting glucose levels. However, it is important to note that glucose levels (as depicted in Fig 1) are already increasing during the late stage of the night. Fasting glucose levels might therefore reflect additional aspects of glucose regulation than nocturnal glucose levels, possibly involving processes around awakening.

To date, the exact mechanism through which the rs7903146-T allele increases serum glucose level, and thus increases the risk of T2D, is not fully understood. Some studies have pointed toward defects in insulin secretion [7,8,14,15], while others argue that TCF7L2-related disruption of β-cell function might be the indirect consequence of primary events in the liver and elsewhere [6]. However, *TCF7L2* overexpression in human pancreatic islets has also been associated with an impaired glucose-stimulated insulin secretion [7,27–29]. Furthermore, the rs7903146-T allele in *TCF7L2* was associated with a reduction of total pancreatic islet number and morphological changes in human islets [28]. However, β-cell-specific *Tcf7l2* knockout mice had similar β-cell function compared with wild-type mice, while increased levels of TCF7L2 in the liver strongly affected glucose metabolism [6]. Liver-specific *Tfc7l2* knockout mice showed reduced production of hepatic glucose, while overexpression of *TCF7L2* showed higher hepatic glucose production than control mice [6]. As a consequence of these contradictive results, it has been proposed that perturbation of *TCF7L2* expression influences metabolic processes in both pancreas and liver [30]. We observed that the mean nocturnal glucose level was higher in participants carrying the rs7903146-T allele in *TCF7L2*. Under normal physiological conditions, plasma glucose is derived from endogenous glucose production in the liver by glycogenolysis in the fasting state under influence of glucagon. However, in the fed state, plasma glucose is derived from nutrients. During this stage, both gluconeogenesis and glycogenolysis are suppressed by insulin. Therefore, these results indicate that the rs7903146-T allele might affect the endogenous glucose production in the liver, as normally no nutrients were taken during the night. In line with this reasoning, it has previously been reported that the rs7903146-T allele in *TCF7L2* was associated with higher basal endogenous hepatic glucose production [7].

In contrast to another study that observed impaired insulin secretion in rs7903146-T allele carriers [7], T-allele carriers had higher fasting insulin levels and a somewhat higher mean overall diurnal glucose level (between 6:00am– 00:00am) in our study. However, the comparison in our study population was not statistically significant. The tendency towards higher fasting insulin levels in rs7903146-T allele carriers might be explained by a compensatory response of the pancreas to the higher influx of endogenously produced glucose associated with this allele. For future studies, incorporating information from both the pancreas and liver in glucose metabolism will likely contribute to a better understanding through which biological mechanism genetic variation in *TCF7L2* affects glucose metabolism.

We additionally showed that the association between rs7903146 and nocturnal glucose levels was partly mediated by body composition (percentage of body fat and body weight). In contrast to a previous GWAS study and a study done in the ARIC study population [31,32], we observed no interaction between rs7903146 and body composition on nocturnal glucose levels, which could be due to our limited sample size. Previously, rs7903146 was shown to be a determinant in intervention studies on weight loss, depending on food composition [33,34]. However, how the interplay between rs7903146, body composition and glucose works is unclear and requires additional studies.

This study has a few strengths and limitations. A strength of our study is the relatively large sample size available with data derived from CGM. However, for a study on genetics, the current study was still relatively small in size. This may have led to a limited statistical power to detect differences between study groups. Also, because of the limited number of homozygous T allele carriers, these had to be combined with the heterozygous carriers. However, Monte Carlo permutation tests gave similar results. The CGM system provides detailed information about many aspects of glucose metabolism, ranging from mean glucose levels during certain times of the day to measures of glycemic variability. With respect to the *TCF7L2* gene, but also genetics in general, the CGM system has not been used before.

In conclusion, within our study population we observed that the rs7903146-T allele in *TCF7L2* was associated with higher mean nocturnal glucose levels but not with measures of glycemic variability. Therefore, this study suggests that the rs7903146 polymorphism in the *TCF7L2* gene affects endogenous production of glucose in the liver.
